# Urea cycle regulation by mitochondrial sirtuin, SIRT5

**DOI:** 10.18632/aging.100062

**Published:** 2009-06-29

**Authors:** Takashi Nakagawa, Leonard Guarente

**Affiliations:** Paul F. Glenn Laboratory for the Science of Aging and Department of Biology, Massachusetts Institute of Technology, Cambridge, MA 02139, USA

**Keywords:** SIRT5, sirtuin, CPS1, mitochondria, urea cycle, deacetylation

## Abstract

Mammalian sirtuins have diverse roles in aging, metabolism and disease. Recently we reported a new function for SIRT5 in urea cycle regulation. Our study uncovered that SIRT5 localized to mitochondria matrix and deacetylates carbamoyl phosphate synthetase 1 (CPS1), an enzyme which is the first and rate-limiting step of urea cycle. Deacetylation of CPS1 by SIRT5 resulted in activation of CPS1 enzymatic activity. Indeed, SIRT5-deficient mice failed to up-regulate CPS1 activity and showed hyper ammonemia during fasting. Similar effects are also observed on high protein diet or calorie restriction. These data indicate SIRT5 also has an emerging role in the metabolic adaptation to fasting, high protein diet and calorie restriction.

Sir2 is a NAD-dependent deacetylase and promotes longevity in many organisms [1]. In mammals, there are seven Sir2 homologues called sirtuins (SIRT1-7), which regulate various biological functions in aging, metabolism and disease. Among of them, SIRT3, SIRT4 and SIRT5 are believed to be localized in mitochondria [2]. SIRT3 is the most characterized mitochondrial sirtuin. SIRT3 interacts with acetyl-CoA synthetase 2 (ACS2) and deacetylates Lys-642 in vitro and in vivo. Deacetylation of ACS2 by SIRT3 up-regulates the acetyl-CoA synthesis activity [3,4]. SIRT3 also deacetylates NDUFA9, one of the electron transport chain complex1 components to regulate ATP levels [5].  SIRT4 ADP-ribosylates glutamate dehydrogenase (GDH) and controls insulin secretion in response to calorie restriction [6,7]. GDH is also deacetylated by SIRT3, but its physiological significance is unknown [8]. These findings show that SIRT3 and SIRT4 directly control the activity of metabolic enzymes in mitochondria and play an important role in energy metabolism. However the function of SIRT5 was unknown.

Recently we reported that SIRT5 is localized in mitochondria matrix and regulates the urea cycle through the deacetylation of CPS1 (Figure 1) [9]. By systemic sub-fractionation of isolated mitochondria from mouse liver, we found SIRT5 was also localized in the matrix fraction, as well as SIRT3 and SIRT4. This finding corresponded with the fact that SIRT5 was cleaved in the N-terminus at a typical consensus sequence recognized by mitochondria matrix peptidase. To identify SIRT5-interacting proteins, we developed *In vitro* SIRT5-Flag affinity purification, and found CPS1 as a SIRT5-binding protein. CPS1 is an enzyme which mediates the first step of urea cycle. CPS1 is reported to be acetylated at multiple lysine residues [10], however the biological function of acetylation was unclear. Using an in vitro deacetylation assay, we revealed that SIRT5 could deacetylate CPS1 in a NAD-dependent manner and this deacetylation increased CPS1 enzymatic activity. Indeed, SIRT5 deficient mice have ~30% lower CPS1 activity compared to wild type mice. During fasting conditions, SIRT5 deficient mice failed to up-regulate CPS1 activity and thereby resulted in hyper ammonemia. Similar results were observed during calorie restriction or a high protein diet.

How does food limitation activates the SIRT5? A previous study showed fasting induced the translocation of NAD biosynthesis enzyme Nampt, to mitochondria and elevated the NAD levels in mitochondria [11]. Although we did not detect the translocation of Nampt to mitochondria in mouse liver, we observed the increase of Nampt protein in the cytoplasm and the increase of NAD level in the mitochondria. We hypothesize that the elevation of Nampt in cytoplasm increase the NMN pool in the cytoplasm and NMN consequently translocates into the mitochondria. In mitochondria, Nmnat3 converts NMN to NAD, the next step in NAD biosynthesis, to activate SIRT5 (Figure 1). However questions remain. For example, does Nampt translocation to mitochondria take place in other tissues? How is NMN incorporated into mitochondria? In mitochondria, ~400 proteins have been shown to be acetylated [10]. However little is known about their biological significance. As SIRT5 is expressed ubiquitously in various tissues, SIRT5 probably has other substrates besides CPS1. In fact, we also identified a few other SIRT5 interacting proteins using same strategy (our unpublished data). Compared to deacetylation, little is known about the mechanism of acetylation of mitochondrial proteins. Is acetylation in the mitochondria enzymatic? If so, how many acetyltransferase are there, and what is the specificity? More studies will be necessary to understand the biological meaning of acetylation/deacetylation interplay in the mitochondria.

**Figure 1.**
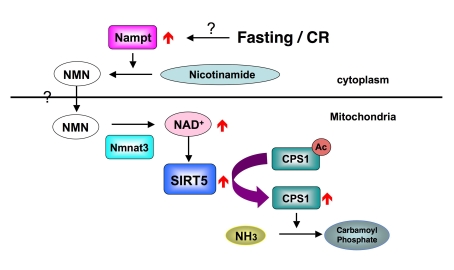
How SIRT5 is regulated during fasting and CR.

Interestingly, SIRT4 ADP-ribosylates GDH, which mediates the oxidative deamination step during ammonia detoxification and represses GDH activity (Figure 2). Our findings show that SIRT5 controls the subsequent step of the ammonia detoxification pathway. Furthermore, OTC, an enzyme mediating the second step of the urea cycle in the mitochondria matrix, was also recently reported to be regulated by reversible acetylation of Lysine-88 [12]. Deacetylation of OTC Lysine-88 increases the OTC enzymatic activity. In our study, no obvious change was observed in OTC activity in SIRT3, SIRT4 and SIRT5 deficient mice under normal conditions. However, a more detailed study under stressed condition, such as fasting or calorie restriction may reveal the roles of mitochondrial sirtuins in OTC deacetylation and the coordination of mitochondrial sirtuins in the ammonia detoxification pathway.

**Figure 2.**
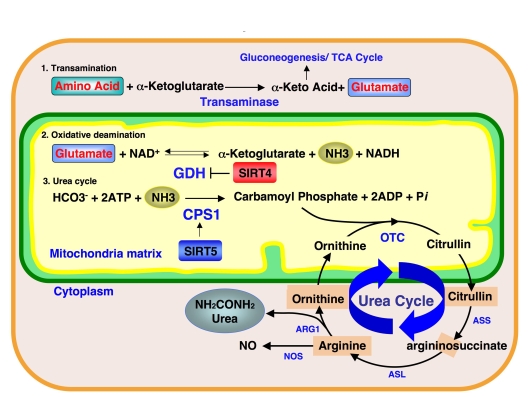
Ammonia detoxification pathway and mitochondrial sirtuins.

Our findings uncovered that, SIRT5 also has a pivotal role in the metabolic adaptations during dietary shifts as well as other sirtuins and co-factor NAD biosynthesis pathway is also the important regulator of these processes. Drugs that activate SIRT5 may have therapeutic value to treat hyper ammonemia.

## References

[1] Guarente L (2008). Mitochondria--a nexus for aging, calorie restriction, and sirtuins. Cell.

[2] Haigis MC, Guarente LP (2006). Mammalian sirtuins--emerging roles in physiology, aging, and calorie restriction. Genes Dev.

[3] Hallows WC, Lee S, Denu JM (2006). Sirtuins deacetylate and activate mammalian acetyl-CoA synthetases. Proc Natl Acad Sci U S A.

[4] Schwer B, Bunkenborg J, Verdin RO, Andersen JS, Verdin E (2006). Reversible lysine acetylation controls the activity of the mitochondrial enzyme acetyl-CoA synthetase 2. Proc Natl Acad Sci U S A.

[5] Ahn BH, Kim HS, Song S, Lee IH, Liu J, Vassilopoulos A, Deng CX, Finkel T (2008). A role for the mitochondrial deacetylase Sirt3 in regulating energy homeostasis. Proc Natl Acad Sci U S A.

[6] Haigis MC, Mostoslavsky R, Haigis KM, Fahie K, Christodoulou DC, Murphy AJ, Valenzuela DM, Yancopoulos GD, Karow M, Blander G, Wolberger C, Prolla TA, Weindruch R (2006). SIRT4 inhibits glutamate dehydrogenase and opposes the effects of calorie restriction in pancreatic beta cells. Cell.

[7] Ahuja N, Schwer B, Carobbio S, Waltregny D, North BJ, Castronovo V, Maechler P, Verdin E (2007). Regulation of insulin secretion by SIRT4, a mitochondrial ADP-ribosyltransferase. J Biol Chem.

[8] Lombard DB, Alt FW, Cheng HL, Bunkenborg J, Streeper RS, Mostoslavsky R, Kim J, Yancopoulos G, Valenzuela D, Murphy A, Yang Y, Chen Y, Hirschey MD (2007). Mammalian Sir2 homolog SIRT3 regulates global mitochondrial lysine acetylation. Mol Cell Biol.

[9] Nakagawa T, Lomb DJ, Haigis MC, Guarente L (2009). SIRT5 Deacetylates carbamoyl phosphate synthetase 1 and regulates the urea cycle. Cell.

[10] Kim SC, Sprung R, Chen Y, Xu Y, Ball H, Pei J, Cheng T, Kho Y, Xiao H, Xiao L, Grishin NV, White M, Yang XJ (2006). Substrate and functional diversity of lysine acetylation revealed by a proteomics survey. Mol Cell.

[11] Yang H, Yang T, Baur JA, Perez E, Matsui T, Carmona JJ, Lamming DW, Souza-Pinto NC, Bohr VA, Rosenzweig A, de Cabo R, Sauve AA, Sinclair DA (2007). Nutrient-sensitive mitochondrial NAD+ levels dictate cell survival. Cell.

[12] Yu W, Lin Y, Yao J, Huang W, Lei Q, Xiong Y, Zhao S, Guan KL (2009). Lysine 88 acetylation negatively regulates ornithine carbamoyltransferase activity in response to nutrient signals. J Biol Chem.

